# Neurotechnology through the lens of users and as a novel field for society

**DOI:** 10.3389/fneur.2026.1842746

**Published:** 2026-07-16

**Authors:** Danesh Ashouri, Ludwig Weh, Vera Borrmann, Christopher Coenen, Eva Maria Kraft, Viviana Loufs, Wenzel Mehnert, Paula Muhr, Oliver Müller, Melike Şahinol, Markus Schmidt, Günter Seyfried, Kai Soedibjo, Ian Erik Stewart, Gregor Wolbring, Sandra Youssef, Thomas Stieglitz

**Affiliations:** 1 BrainLinks-BrainTools Center, University of Freiburg, Freiburg, Germany; 2 Laboratory for Biomedical Microtechnology, Department of Microsystems Engineering (IMTEK), University of Freiburg, Freiburg, Germany; 3 Humboldt-Universität zu Berlin, Berlin, Germany; 4 Human-Technology Interaction Lab, Department of Neurosurgery, University of Freiburg - Medical Center, Freiburg, Germany; 5 Institute for Technology Assessment and Systems Analysis (ITAS), Karlsruhe Institute of Technology, Karlsruhe, Germany; 6 RAUM für TANZ KG, Vienna, Austria; 7 Brandenburg University of Technology in Cottbus-Senftenberg, Cottbus, Germany; 8 Austrian Institute of Technology, Vienna, Austria; 9 Digital Design Department, Brand University of Applied Sciences, Hamburg, Germany; 10 Social Studies of Science and Technology, Technische Universität Berlin, Berlin, Germany; 11 Department of Philosophy, University of Freiburg, Freiburg, Germany; 12 Science, Technology and Society (STS), Orient-Institute Istanbul, Istanbul, Türkiye; 13 Biofaction KG, Vienna, Austria; 14 Department of Neuroscience, Max Delbrück Center for Molecular Medicine (MDC), Berlin, Germany; 15 Department of Biology, Chemistry, and Pharmacy, Freie Universität Berlin, Berlin, Germany; 16 Department of Community Health Science, Program in Community Rehabilitation and Disability Studies, Cumming School of Medicine, University of Calgary, Calgary, AB, Canada

**Keywords:** neurotechnology, brain-computer interface, embodiment, neuroethics, society, arts, technology assessment

## Abstract

Successful and responsible innovation in neurotechnology requires clear ethical priorities and a deep understanding of individual and societal needs as well as public concerns. Recent cases of consumer exploitation, misleading claims, and inadequate patient aftercare reveal critical gaps in current practices and underscore the urgent need for more ethical, transparent, and user-centered engagement in this rapidly developing field. This study focuses on four complementary domains: (1) neuroethics and embodiment; (2) the cultural embedding of neurotechnologies; (3) art and culture in relation to neurotechnology; and (4) human enhancement, technovisions, and sociotechnical imaginaries. Across these domains, the manuscript explores user and societal perceptions, highlighting often overlooked asymmetries in communication between scientists and entrepreneurs and those who ultimately receive research outcomes in the form of products. Drawing on the authors’ multidisciplinary expertise and a synthesis of the relevant literature, the manuscript outlines a possible foundation for developing more balanced, inclusive and symmetric communication formats that empower stakeholders regardless of status or expertise. Integrating insights from neurotechnology with applied ethics, the humanities, social sciences, technology assessment and the arts, this work seeks to contribute to a broader understanding of the societal and individual impacts of emerging neurotechnologies and to support the protection and empowerment of users by prioritizing their needs.

## 1 Introduction

**Figure 1:**
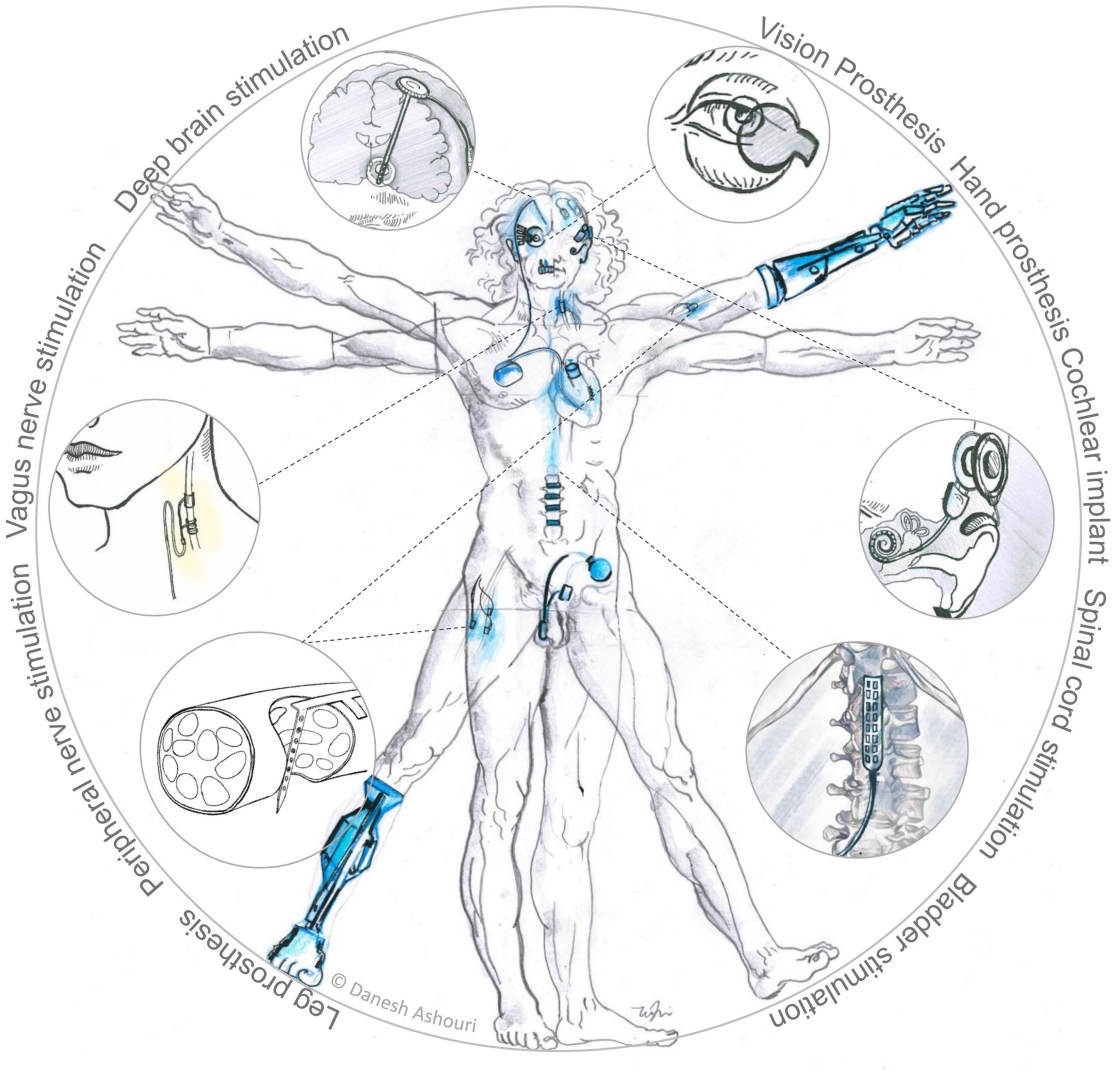
Exemplary application fields in neurotechnology.

Neurotechnology creates a communication link between the nervous system and technological devices (1). Besides a broad range of prosthetic and therapeutic uses (Figure 1), its applications extend beyond the medical domain into the commercial, recreational, and even military sectors (2). The significant economic growth in this area in recent years is a testament to the surge in use cases of neurotechnology (3), generating a market value of USD 12.82 billion in 2022 and projected to reach approximately USD 38.17 billion by 2032 (4). The global neurostimulation devices market alone was valued at USD 6 billion in 2021 and is expected to reach USD 14 billion by 2030 (5).

Enhancing human capabilities seems to be a growing trend in the novel uses of neurotechnology (2). In an early study commissioned by the European Parliament, human enhancement is described as “modification aimed at improving individual human performance and brought about by science-based or technology-based interventions in the human body” (6). Such enhancements may be used to restore impaired functions (e.g., sensory or limb prosthetics or cognitive functions that help an Alzheimer’s patient to remember) or to enable a non-biologically typical function, such as the ability to control machines without physical interaction (7).

Despite the continuous growth of neurotechnology, both economically and in terms of its applications, the assessment of the field, and, correspondingly, its societal embedding and regulatory oversight, still lag behind (7). Such embedding could perhaps be strengthened by focusing to a greater extent on the individuality of user needs, by fostering interactions between the various stakeholder groups and their interests, and by addressing the broader societal relevance of neurotechnological progress (8). One important development toward achieving this is the UNESCO declaration on “ethics of neurotechnology” which was released in November 2025 and now calls for its principles to be incorporated into hard law at the national level, a challenge that requires new perspectives on the use cases of neurotechnology (9).

Existing shortcomings in ethical and regulatory standards are evidenced by instances of inadequate patient care and market failure (10). Insufficient ethical guidelines may contribute to gaps in sustained responsibility toward patients involved in clinical trials within the medtech industry (11). This can result in situations where patients are left with implanted devices without clear aftercare plans once the trial has been completed and data has been collected (12, 13). One example of this is Second Sight, a manufacturer of retinal implants, which stopped providing support for its products in 2019 and almost went out of business in 2020 (14). This left users of the implants with almost no support or guidance until 2023 (15), when the company Cortigent was set up using Second Sight’s technologies, giving rise to hopes that even if the system itself is not developed further, ARGUS II users could at least be provided with the necessary spare parts and maintenance (16).

As regards non-medical uses, whether it is non-implantable or implantable, the situation remains inadequate despite recent efforts to strengthen the regulatory framework (17, 18). The deficiencies in the current regulatory process allow profit-driven companies to conduct self-funded research without their products being externally reviewed (19, 20). This lack of oversight can lead to the dissemination of misleading or unsubstantiated claims, resulting in lawsuits and fines, e.g., online games that falsely claim to help users perform better at work and school and avert cognitive deficits associated with serious mental disorders (21, 22).

In addition, a widening gap between technological advancement and public understanding, especially in the context of ‘neuro-hype’ (23, 24), could potentially enable exploitative practices in the absence of ethical measures. Even commercial enterprises that do not act in bad faith may nonetheless fall short of best ethical practice, due to weak enforcement of existing regulations and the tendency of emerging technologies to encourage developers to defer ethical concerns to a hypothetical future problem (25). This could even leave them with little alternative given the lack of obvious platforms for discussing and developing guidelines. To address the current environment of misunderstanding and miscommunication (26, 27), it is crucial to find strategies that help refine and restructure the regulatory guidelines in such a way that the necessary perspectives can be incorporated.

As suggested by Yuste and colleagues (28), privacy, identity, agency, and equality are the four priorities that should be preserved and respected when developing advanced neurotechnologies. However, the exact strategies of such implementations are complex and require communication as well as collaboration across many disciplines together with researchers and developers in the field of neurotechnology. Such a multifaceted approach broadens developer perspectives by considering different insights that do not always overlap. When defining invasiveness, for example, a developer may limit the definition to physical intervention. From an ethical standpoint, however, invasiveness can also refer to emotional, lifestyle and physical interference (29), which demonstrates how reconciling different points of view in the understanding of a common problem can help strengthen patient-centered and socially diverse perspectives.

This paper offers a glimpse into non-mainstream perspectives on neurotechnology that reflect the field’s creative diversity and its current and growing societal significance. The hope is to bring about a deeper understanding of the specificities of individual uses of neurotechnical devices and the diversity of user experiences. Additionally, it supports efforts to shape neurotechnology by empowering users through participatory approaches involving inter- and transdisciplinary communication. Such engagement can help promote a more accurate representation of complex scientific facts, help demystify neuro-hype, and foster socially beneficial applications. Accordingly, the paper integrates perspectives from neurotechnology, neuroethics, science and technology studies (STS), technology assessment, futures studies, social sciences, disability studies, ability studies, and the arts in order to discuss the prospects for technology development from different viewpoints and to re-examine the future direction of this dynamic and rapidly evolving field.

We believe that such collaborative efforts are essential for bridging the existing gaps in the field of neurotechnology, serving as a means to prioritize patients’ needs, strengthen public trust and ensure the responsible and sustainable growth of the neurotechnological developments.

## 2 Neurotechnology: hopes, limitations and speculations

**Figure 2:**
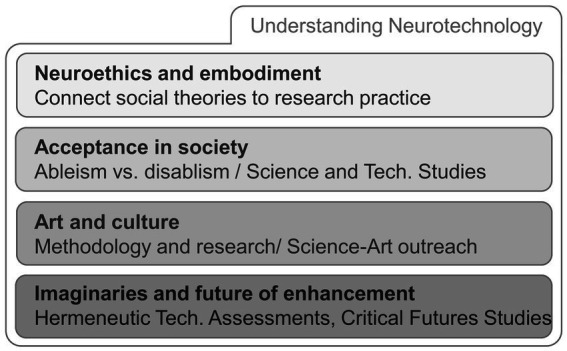
Argumentative perspectives for the ethical consideration of neurotechnology within the scope of the paper.

In four complementary sections, this paper examines neurotechnologies from inter- and transdisciplinary perspectives to better understand their ethical and societal implications. It highlights patient- and consumer-centered insights that, although increasingly recognized, remain insufficiently embedded in widespread research and development practices (Figure 2).

In the argumentation, we review core assumptions of phenomenological and postphenomenological theory that underpin embodied approaches to studying neurotechnologies (30). Phenomenology is a philosophical approach that studies how things appear in our lived experience, focusing on the structures of consciousness, perception, and the embodied, first-person perspective through which we experience the world (31). It emphasizes *how* we experience, rather than *what* we experience. These theoretical premises translate into arguments raised by interdisciplinary fields such as medical sociology, anthropology or STS, emphasizing that the embodied appropriation of neurotechnologies within different social status groups may change individual and collective notions of physical and self-identity, for example by affecting ability norms and privileges (32, 33).

Ensuing examples of science-art initiatives and hermeneutic technology assessment can show, from a critical and participatory science communication perspective, how such research can account for changes in social narratives following the introduction of novel neurotechnologies.

Building on these approaches, the paper outlines concrete action points for incorporating ethical reflection and societal awareness within the translation of basic research into applied innovation in neuroengineering and neurotechnological development. By underscoring the importance of science-society interfaces, we advocate strategies that promote the ethical integration of contemporary neurotechnology, approaches capable of anticipating and managing both the positive and potentially problematic sociocultural side effects of neurotechnological interventions in preventive, therapeutic and human enhancement domains.

### 2.1 Neuroethics and embodiment: translating phenomenology and science and technology studies into research and technology development

Advanced neurotechnologies can potentially transform the understanding of the brain, perceptions of the self, sense of agency and interactions with the world (34). Their use raises important ethical and social concerns and questions about their potential impact on embodiment. Examples include the development of brain-computer interfaces (BCI) and neuroprosthetics that enable direct communication between the nervous system and external devices, in the form of either non-invasive or invasive brain-computer interfaces (iBCIs) (35). While the topic of embodiment has been addressed in the context of BCIs (36) and neuroethics in general, we advocate for including more contemporary phenomenological perspectives of lived experience in the discourse, given the richness of the phenomenological tradition in engaging with these thoughts (37, 38).

Neurotechnologies could potentially blur the lines between human and machine. In particular, this could fundamentally alter the relationship between the body and its environment, e.g., regarding the way individuals experience their own bodies, given that sensory inputs are mediated through an external device. This could also lead to a felt sense of bodily disconnection and alienation, prompting questions about how such changes might affect not only the quality of an individual’s experience but also their sense of self or agency (39–41).

The potential impact of neurotechnologies on the lived-experience and the lived-body including the potential benefits as well as the associated risks can be captured in its multifaceted nature through phenomenological approaches. The phenomenology of the lived body, emphasizes the importance of embodiment and the lived-body in shaping human experience and perception (42). Here, perception is considered to be always embodied, and being embedded in the world is constitutive for any bodily experience. Thus, corporeality mediates and shapes the way one perceives and interacts with the world (38, 43–45). Contemporary phenomenology has increasingly been integrated with cognitive sciences, (neuro)psychology, psychiatry and neurosciences (46–49), allowing for interdisciplinary explorations such as neurophenomenology (50), feminist (51), queer (52, 53) and crip phenomenology (54, 55), or postphenomenology (56, 57)_._ Postphenomenology focuses on how technologies mediate human experience and perception and studies the relations between humans and technologies, showing how technology shapes the way we experience, understand, and act in the world. A postphenomenological perspective on embodiment and technology, combined with perspectives of feminist and critical phenomenology, provides a particularly valuable lens through which these concerns and their neuroethical implications for neurotechnologies can be explored (58). This perspective emphasizes that technological development is always socially, culturally, and historically situated, highlighting how power structures and normative frameworks influence the design, deployment, and impact of neurotechnologies, how they are used and also what they represent. By foregrounding these dimensions, such perspectives complement classical phenomenological analyses of embodiment and provide critical insight into how neurotechnologies shape human experience, agency, and ethical responsibility.

As an example, the development of neurotechnologies to treat mental health conditions might be based on biased assumptions about gender, race and other social identities, which might lead to biased development paths or unequal access to these technologies. This could potentially result in harmful outcomes, particularly for individuals with disabilities (59). For example, if such marginalized groups were disproportionately affected by the technologies’ impact on their individual autonomy and identity, or if this could raise concerns about privacy and surveillance (60, 61) given that neurotechnologies may provide unprecedented access to individual data. In this context, critical feminist, queer, crip and postphenomenological perspectives constitute a useful tool to assess the impacts of neurotechnology on embodiment and lived experience, especially with a focus on societal norms that are inscribed in technologies, both explicitly and implicitly. Understanding the complex interplay between bodies, experiences and interactions with the world can also help to identify potential ethical concerns.

Translating aspects from phenomenological theory to applied research practice in neurotechnology may challenge dualistic mind–body assumptions underlying some commercial developments and marketing of products for self-improvement or human enhancement (62). Furthermore, it demands a review of the related technovisions or sociotechnical imaginaries in social discourse and the adoption of more integrative, applied and solution-oriented ways of researching emerging neurotechnologies (63). Such research practices have deeply transformative implications for traditional research designs. The phenomenological plea for ‘(re)embodied’ ways of knowing resonates with post-normal demands for research adhering to social discourse and real-world problem-solving. This can turn the research designs into more critical, relational, autoethnographic or post-normal approaches.

For neuroethics “concerned with ethical, legal and social policy implications of neurosciences” (64), transdisciplinary research designs can integrate multiple perspectives for public trust-building as a crucial element of successful practical research (65) and as a means to improve truth, belief and justification conditions of scientific knowledge production (66). This can improve the social acceptance and integration of neurotechnology development. On a path toward problem-based and solution-oriented research, social discourse as an integral part of transdisciplinary paradigms actively involves non-scientific audiences in participatory, democratic forms of knowledge production and uncertainty absorption at science-society interfaces (67).

Such critical and discourse-based approaches to technology development are shaping fields such as STS and technology assessment (TA). Considering emerging neurotechnologies from these perspectives, specific research frameworks like Responsible Research and Innovation (RRI) have been established, which invite researchers to consider emerging neurotechnologies from a (post)phenomenological, relational materialist perspective for their socially integrated, beneficial and desirable development, dissemination and use (63). Box 1 describes an example project which implemented these criteria.

### 2.2 The cultural embedding of neurotechnologies: ableism vs. disablism and DIY neuroculture

As neurotechnologies become increasingly accessible and prevalent, their impact on our understanding of ourselves and our place in society has been the subject of many projects around the world that aim to enhance our understanding of the human brain (68). These initiatives, however, focus primarily on the function of the brain and often overlook the socio-cultural impacts that neurotechnologies may have on individual and collective identities as well as our place in society and on the ways these developments are addressed through public engagement efforts (69).

BOX 1 **Project overview – FUTUREBODY**

The EU ERA-NET NEURON project ‘FUTUREBODY—the Future of the Body in the Light of Neurotechnology’ (70) researched the ethical, legal and social aspects (ELSA) of emerging neurotechnologies. Besides addressing theoretical considerations about (1) surgical interventions augmenting bodily functions, (2) non-invasive neurotechnologies and (3) brain-computer interface technology, the project conducted a number of transdisciplinary research and science communication formats using science-art methods. Fostering discourse at science-society interfaces – e.g. via a film festival and other discussion formats – the project assessed desirable sociotechnical images of neurotechnologies and enabled participatory reflection and mutual learning between academic and varied non-academic stakeholder groups.

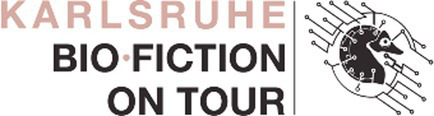

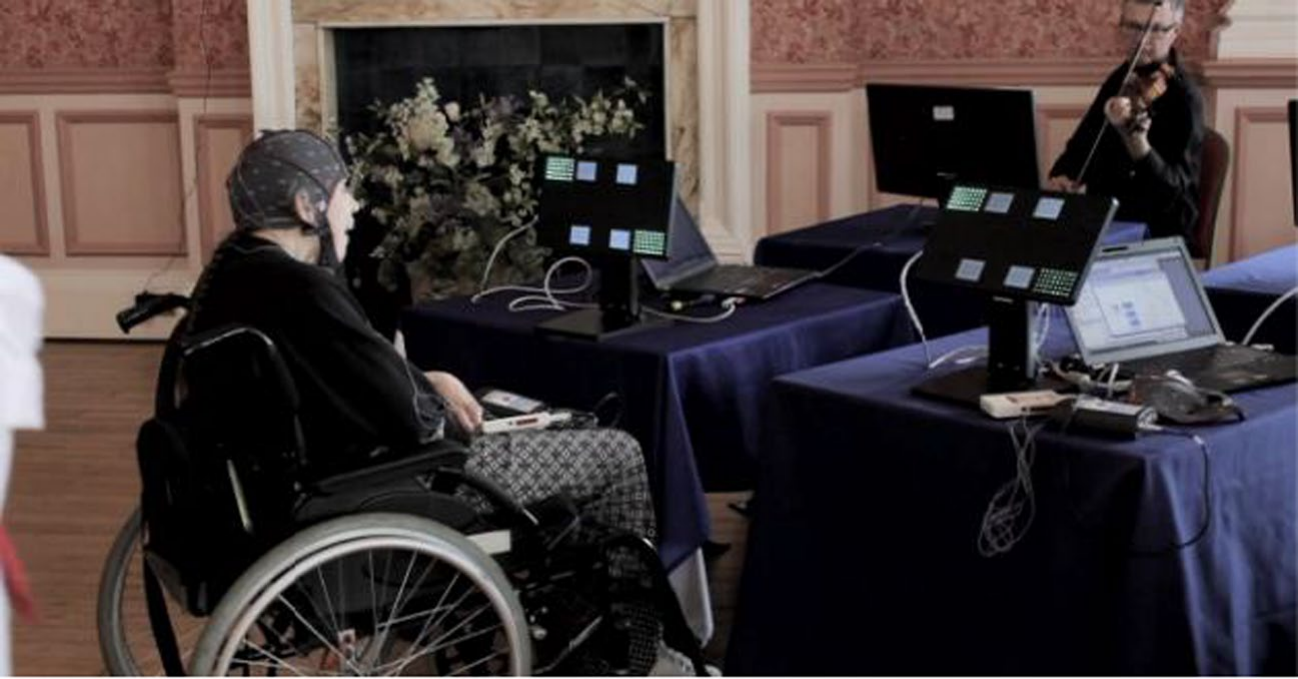

The format of BIO·FICTION (71) film screenings and public debates created an easy-access set-up that has been replicated in several places around the world.

Screenshot from the short documentation film “Paramusical Ensemble” (72). Image credit - Tim Grabham ‘Paramusical Ensemble’ 2015.

The film “Paramusical Ensemble” was one of the winners of the 2019 BIO·FICTION Science Art Film festival, organized as an inter- and transdisciplinary exchange platform within the FUTUREBODY project (70). Among other winning and jury-selected films, it was presented around the world either physically—like at EFFEKTE-Filmfestival in Karlsruhe 2021—or online during a “film world café” discussion series.

These events invited non-academic audiences to join discussions about the hopes, fears and doubts regarding neurotechnological possibilities and their implications for human corporeality. The film festival serves as a suitable medium and agora for combining art and science via curated film screenings followed by public debates. In this way, it enables a lay audience to participate in a discussion about ethical issues of neurotechnology-inspired futures.

In this context, it is crucial to recognize and consider concepts of ableism and disablism, driven by the disabled people’s rights movement, which also challenge the ability norms and privileges associated with emerging neurotechnologies. The bottom line here is based on one’s body–mind ability to have a good life and on the reassuring sense of being at ease with one’s abilities (69, 73, 74).

Over 25 different ability-judgment-based theoretical frameworks have been developed in the four strands of ability studies (75–77), including technology-specific concepts such as techno-ableism, techno-disablism, techno-poor disabled, and techno-poor impaired (73, 78). Such concepts focus on the investigation of ability-based expectations, judgments, norms and conflicts. These ability-based concepts are not only relevant to disabled individuals who are at risk of being labeled as defective and of being seen as targets for neuroenhancing/ability-changing products. They are also relevant to individuals who are regarded as ‘normal’, and to non-neuroenhanced people who, in a more technologised future, may be conceived of as disabled. Individuals who are judged as ability-deficient or disabled often accept these labels and internalize such ability judgments by others, and with that may also internalize negative treatments they experience as a result of that ability judgment (internalized disablism) (79).

This becomes particularly crucial in the case of neurotechnologies such as implantable BCIs, which aim to help people affected by stroke, spinal cord injury (80) or amyotrophic lateral sclerosis (ALS) and to enable them to interact with their environment (81). A review showed that invasive BCIs are almost exclusively addressed within the framework of disabled people as patients, whereas non-invasive BCIs such as those used for gaming are rarely addressed in relation to disabled people (33).

It is important to note that our understanding of the human subject can be changed not only by the adoption of neurotechnologies but also by their potential to transform the lives of differently abled individuals with increasing accessibility to technologies such as BCIs (33). However, the use of neurotechnologies for self-enhancement can also create pressure to constantly optimize and improve the human body and mind, potentially leading to unrealistic expectations and a never-ending quest for perfection.

When examining the appropriation of neurotechnologies, it is thus crucial to delve into the sociocultural contexts underpinning their development and usage. Cultural beliefs about the mind and body may vary and can influence the acceptance of or attitudes toward certain types of neurotechnologies. Additionally, the attitudes of different social groups toward healthcare, science and technology can significantly impact their access and willingness to use neurotechnologies. Within the deaf community, for example, the varied opinions about and experiences of cochlear implants (82) reflect a broader commitment to cultural and linguistic identity rather than to a mere technological fix (83) for a disability. As another example, neurological conditions such as autism or attention deficit hyperactivity disorder (ADHD) are not inherently negative, nor do they need to be cured (as the term ‘neurodivergence’ signals). Discussed within the topic of neurodiversity, society might perhaps embrace and accommodate these differences as being all part of human variation and combined with a kind of “neurological self-awareness” (84). In this light, the promotion of neurotechnologies that aim to “cure” these conditions can be viewed as problematic.

To explore the complexities of neurodiversity and society’s potential to embrace neurological differences, it is essential to consider the emerging trend of do-it-yourself (DIY) neuroculture. This phenomenon, embodied by non-professionals and neurohackers experimenting with neurotechnologies, reflects a grassroots movement that operates outside traditional medical settings and aligns with the broader dialog on neurotechnological interventions (85). Through online platforms and social media, members of this community share their experiences, knowledge and expertise, creating a novel form of knowledge production and dissemination. Parallels exist with drug use, particularly psychedelics, as both can be seen as tools for personal enhancement that demand consideration of safe and legal use. However, DIY neuroculture’s innovation in democratizing scientific knowledge and technological innovation raises critical safety and ethical concerns, especially regarding the risk of untrained individuals inadvertently exposing themselves and others to harm and unintended consequences. This underscores the need for public education about the potential benefits and risks of neurotechnologies, encompassing medical applications and the ethical and social implications of non-medical use.

Such cultural aspects of neurotechnology – the cultural meaning of neurotechnologies and the process of technological appropriation by different communities – are still too often overlooked in both the communication between developers and companies and the ethical discourse on the technologies. In this context, we emphasize the relevance and potential of creative approaches when it comes to fostering a better understanding of the cultural and societal dimensions as well as their societal embedding.

### 2.3 Art and culture, with or versus neurotechnology

Since the 1960s, when artists began exploring the fusion of neuroscience and evolving neurotechnologies, neuroart has surged in prominence. This has been reflected in a growing number of academic publications and artistic projects, and in the increasingly diverse roles that neuroart has assumed (86). Neuroart, also known as brain art and neurotech art, refers to artworks that deal with the human brain, may draw on neuroscientific knowledge and may deploy neurotechnology.

In the following section, we highlight examples from a range of neuroart projects to underscore the societally relevant technical and conceptual advances in neurotech applications that have been made outside the context of public or commercial industrial research and development. The breadth and depth of neurotechnology-related discourse in art represent a potent source for transdisciplinary development, from which current neurotech and artistic research alike could benefit.

In this section, we focus on three prominent roles of neuroart: as a medium for (science) communication, as therapeutic engagement and as an epistemological framework. It is important to note that it is not our intention in placing this focus to reductively frame neuroart as a mere tool for fulfilling these roles, rather we aim to trace the diversity of neuroart’s cultural and societal implications through its capability to reach broader audiences. Moreover, to do justice to the non-instrumental character of neuroart, we will show that the thread connecting the three different roles discussed here is neuroart’s explorative potential to challenge the current limits of scientific knowledge and state-of-the-art neurotechnology applications.

The communicative role of neuroart is understood to be within the context of science communication and its various modes of operation, ranging from top-down information transfer to more interactive non-hierarchical approaches, termed dialog models (87, 88). Neuroart addresses the fact that transparent exchange with a well-informed public is crucial for responsible neurotechnology development. Currently, a large gap exists between the neuroscientific understanding that underpins neurotechnology and the level of public education and discourse.

In an attempt to bridge this gap, many scientific institutes and neurotechnology companies are increasingly incorporating art projects in their public campaigns. For example, the University of Barcelona organizes an annual science communication project titled ‘NeuroArt’ to encourage participants to create neuroscientifically inspired artworks (89). In the private sector, Blackrock Neurotech, a producer of advanced BCIs, exhibited digital artworks created by patients with paralysis who had received a BCI implant. The resulting exhibition showcased the empowerment brought about by BCI implant technology, albeit without acknowledging the therapeutic aspects of art itself (90). The communicative value of such art projects lies in their ability to engage broader audiences through esthetic and social values, while educating the public in non-reductive ways about largely unfamiliar and often complex neuroscientific concepts and state-of-the-art technologies. In doing so, such art projects make neuroscience more accessible, tangible and engaging for non-specialists.

While valuable, this form of communication functions mostly in one direction, and plays into scientists’ preference for and prioritization of educational science communication goals rather than fostering a more active dialog with non-specialist audiences (91, 92). This distinction reflects broader historical and current debates within science communication regarding the limitations of one-way information-transfer (“deficit”) models and the potential of more dialogical and participatory approaches to public engagement (93–96).

In contemporary science communication approaches, communication is understood not merely as the transmission of information, but as an experiential and emotional process of dialog, participation, and shared meaning-making (97–99).

NeuroArt could fulfill a participatory function by facilitating multidirectional communicative exchanges among researchers, artists and the public that allow all parties to actively learn from the others and jointly co-create and negotiate social and cultural meanings of neurotechnology and neuroscientific concepts. For example, the NEUROART project at King’s College London pairs neurodivergent artists with neuroscientists who conduct research on topics related to neurodiversity (100), engaging them in a mutual dialog that results in the creation of artworks.

It is this more integrative, participatory approach to the communicative role of neuroart (101) that we view as particularly promising and relevant to future developments (further examples exist (102–105))_._

Importantly, neither in the well-established (top-down) nor in the emerging (multidirectional) approaches to neuroart as a means for communication is art used to passively convey a fixed message. Instead, the potential of such art projects lies in their ability to actively engage both specialist and non-specialist stakeholders in the act of exploring the potentials and meanings of neurotechnologies.

Beyond its communicative capacity, art has a second role in the sense that it can empower its practitioners through a *therapeutic function*, as formalized by psychology’s recognition and implementation of art therapy (106). In the context of neuroart, the productive interplay of neurotechnology, science and artistic exploration can yield a cross-fertilization that may help patients express themselves creatively regardless of their mental or physical condition. For example, the documentary film ‘Paramusical Ensemble’ (see Box 1 above) engagingly demonstrates how EEG head-wear enables severely motor-impaired patients to choose musical phrases, thus interacting with a live string quartet and actively shaping a concert in real-time (72). Similarly, Dutch designer Anouk Wipprecht’s project ‘Agent Unicorn’ seamlessly integrates gaming aspects with a medical application. Wipprecht designed a BCI headpiece in the shape of a unicorn’s horn to aid nonverbal communication and everyday life for children with autism and ADHD (107, 108). Her practical work in turn inspired medical engineering company g.tec to host regular brain-computer interface hackathons that bring together engineers, programmers, designers and artists, providing an opportunity to study current and future developments and possibilities of BCI (109). In another interdisciplinary project, dancers and choreographers Monica Gillette and Mia Haugland Habib invited individuals with Parkinson’s disease to collaborate with professional choreographers and scientists from neurotechnology and brain research (110). As equal members of a joint learning process, the participants deployed an artistic research method to explore dance/choreography in movement control and manipulation while focusing on various themes, such as balance, identity, interactivity and embodiment. The intended empowerment and possible therapeutic effects of these diverse art projects integrate patients’ personal experiences, social connections and scientifically-based insights, as in the case of BrainDance, where people with movement disorders were active participants rather than passive recipients of therapy and the common abbreviation PD stood not for Parkinson’s disease but for Parkinson’s dancer.

The third role of neuroart, as an epistemological framework, is reflected in the development of collaborative forms of creative knowledge production that interweave science and art (111).

Neuroart offers the flexibility to intertwine empirical accuracy with imaginative scenarios, constructive criticisms and subjective experiences. Art projects employing such an approach may discuss and challenge not only the seemingly straightforward scientific validity of existing neurotechnologies but also the implicit underlying principles and broader sociocultural implications of their applications (112).

For example, artist and research duo Karen Lancel and Hermen Maat’s ongoing project ‘EEG Kiss’ can offer some deeper insights into the epistemological underpinnings of various aspects of scientific practice. In ‘EEG Kiss’, participants assume roles as hosts, actors or observers with a view to exploring how EEG technology can visualize and be used to objectify and quantify intimacy and the mind. It deconstructs traditional kissing to create a “communal kiss” facilitated by a BCI, envisioning a future where intimate relations and mirror-processes are influenced by technologies like BCIs. The shared, collective interpretation of data not only takes issue with the perceived objectivity of EEG data (“brainwaves”), but also questions the sociocultural contexts of scientific data and the interests and values of those who produce and analyse it, be they scientists, artists or participants in the audience (113).

Another pertinent example of neuroart as an epistemological framework is Mindaugas Gapsevicius’ participatory event titled ‘You and Me, You and I’, during which participants wear elaborately designed jewelry, shoes and headwear with integrated EEG and transcranial direct current stimulation (tDCS) electrodes working in a loop (114). These devices enable the participants to explore otherwise imperceptible inter-individual connections via neuroart-mediated experiences of telepathy and empathy.

Although such imaginary scenarios remain “merely” speculative from a traditional scientific perspective, they can broaden the scope of critical discourse by inviting audiences to ask how future technologies might alter our environment and influence the ways we will communicate with one another.

By including embodied and subjective interactions with and reflective negotiations of neurotechnologies, artistic explorations create novel, immersive sensory spaces that, although facilitated by neurotechnologies, explicitly question any mechanistic understanding of such technologies. Thus, instead of offering a passive reproduction of the already established perspectives on neurotechnologies, neuroart in its diverse roles explores the alternative applications of these technologies and critically examines their future societal implications in terms of both possibilities and limitations.

### 2.4 Human enhancement: interpreting technovisions, sociotechnical imaginaries and images of the future in neurotechnology

Culturally shaped technovisions or sociotechnical imaginaries (115) are determining how people perceive, judge and relate to emerging technologies (116, 117). Interdisciplinary fields such as STS, TA, sociology and anthropology of technology, design or engineering have analysed these visions as images of the future to ascertain which inherently normative statements they make about desirable sociotechnical systems and forms of human-machine interaction (118). From a critical-reflexive and pragmatist perspective (119), researchers have sought to identify and co-create alternative technovisions, e.g., in futures studies, speculative design or art-based research settings. Raising ethical questions in participatory formats at science-society interfaces, they have looked at predominant technovisions or imaginaries voicing personal feelings and expectations in society. These considerations have especially focused on emerging neurotechnologies and artificial intelligence because of their potential to alter human corporeality toward augmented, enhanced or transhuman visions of the human body (120–122). The discourse-based analysis of technofutures, e.g., as vision assessment, has been described as a hermeneutic approach (123).

Research into brain-computer interfaces and, more generally, neurotechnologies has spawned a variety of visions of sociotechnical futures (124) of the human body and how to enhance its capabilities. These visions range from utopian promises of overcoming the human body and its alleged deficiencies to dystopian fears of surrendering humankind to technological progress. They therefore attribute not only capabilities, but also hopes and fears to neurotechnologies long before these technologies have actually come into use and long before their potential has been explored. Examples include interpersonal communication without the use of language (125), the fusion of humans and machines as the next stage of evolution, as promoted in pop culture and mainstream media (126), and the dystopian warning of a neurocapitalistic society separated by a technology gap between those with access to the alleged enhancing features of the neurointerface and those without (127–129). Such visions have been described as being “driven successfully by a scientifically enacted futurism, making neuroscience a poster child of venture capitalism” (130). In emerging neurotechnologies, sociotechnical imaginaries as normatively charged images of techno-futures appear in different forms and media contexts, ranging from formulated texts (e.g., research programs, press, fiction) to materially articulated prototypes, images, films and audio-visual clips, company presentations, TED talks or other forms of science-communication. To assess their contribution to social discourse and the regulation of neurotechnologies, their underlying narratives can be analysed from a critical media perspective (131). In these media, new forms of neurotechnology are associated with images of humans and society in the future, often in a purely hypothetical and speculative manner. They often follow the if-and-then pattern (132), stating that the technology might be capable of a certain function (e.g., enhancing the human body, correcting alleged dysfunction, telepathy, mind-uploading etc.) which would then bring about a certain change in human society for better or worse (e.g., becoming more efficient, evolving to become an interstellar species, achieving immortality, collective mind control etc.). One example of such an if-and-then statement would be: “If it became possible to create a direct interface between brains and machines, this research would threaten to invade privacy when machines are used to read human minds” (132).

Such sociotechnical imaginaries are often also reflected in art-science approaches, such as neuroart projects (see Section 2.3 above). Merging neuroscience, neurotechnology and artistic curiosity, neuroartists explore novel approaches to perception, cognition and senses, delving into neurotechnology’s core elements and challenging them at both the hardware and software level.

The science-fiction short films ‘The Auxiliary’ by Frédéric Plasman (133) and ‘Perfectly Natural’ by Victor Alonso-Berbel (134), for example, present artworks which perform social critiques of imagined future developments in neurotechnology and human enhancement.

More hands-on examples include performance artists or biohackers who engage in DIY practices (see also Section 2.2. above) and use their own bodies to physically feel and translate sensations transmitted by neurotechnologies to their audiences – from Neil Harbisson’s color-to-sound and WiFi-enabled ‘Cyborg Antenna’ implanted to his skull to Moon Ribas’ ‘Seismic Sense’ foot implants which allowed her to sense earthquakes around the world and formed the basis for some of her performance work (85). The general nuances associated with non-scientific, non-artistic neurohackers experimenting with DIY practices have also been explored in Section 2.2. While neurotechnologies are generally viewed as a potential vehicle for human enhancement, their artistic exploration often blurs the line between different perspectives and realities, leading to possible alternative realities and narratives and overcoming seemingly straightforward visions of future applications.

Although such visions are necessarily speculative by nature, they are an essential part of the discourse on human enhancement (135, 136): they evoke different ideas about how neurotechnologies may change the social reality and thus influence discourse on definition and regulation of emerging technologies in the future (137, 138). As early research into the sociology of expectations and into the discourse on emerging technology has shown (116, 139), visions play an important role as they guide the technology’s development and the actions of the developers. This makes it important to analyse and understand these visions and the hopes and fears associated with their cultural background, as well as their strategic role in the discourse. Accordingly, vision assessment deciphers the societal meaning attached to neurotechnologies by analysing the cultural imaginaries enclothed in the circulating visions.

Such hermeneutic analyses reflect on the current hopes and fears inherent to contemporary cultures and societies. In turn, this enables the investigation of alternative images of the future and ultimately promotes democratic debate about progress in scientific-technological developments (140). The aim of hermeneutic analysis is to reflect contemporary beliefs about the contemporary techno-futures by deciphering the inherent attributions of social meaning, the underlying values, the construction of the vision itself, as well as the discursive reaction and their impact on development (141).

An important aspect here is the materiality of neurotechnology and its relationship to the narratives about it. While approaches such as actor-network theory (ANT) have long highlighted the significance of the interrelationships between humans and artifacts, phenomenological approaches offer perspectives on the relationship between technology and the lived body (i.e., the body one is, as opposed to the body one has). And approaches such as narrative bioethics – where phenomenology and hermeneutics can overlap (142) – and hermeneutic technology assessment incorporate narratives from patients or other (potential) users regarding this techno-corporeality. Compelling narrative testimonials, such as that of the renowned scholar Helmut Dubiel (143) – who, as a Parkinson’s patient, wore a deep brain stimulation (DBS) implant – have inspired technology assessment studies (6), including reflection on what it means to be human in an era of highly technologised bodies.

A critical perspective on neurotechnological technovisions can help to contextualize phenomena like ‘neurohype ‘or ‘neuroenhancement‘, understood here as exaggerated expectations, promotional narratives, or overly optimistic claims surrounding emerging neurotechnologies. Such perspectives can prevent the misattribution of capacities and potential impacts to these technologies and instead direct attention to questions such as: What is motivating and driving the speculative developments under consideration? Which individual rights may be affected? Which images of humans, nature and technology are being formed and how are they changing? Which anthropological issues are involved? Which normative models for society are implied in the future projections? This makes hermeneutic analysis a valuable tool for critical-reflexive analysis of the various debates surrounding emerging technologies (144).

To address the gap between rapidly developing and spreading neurotechnologies and the need to understand and regulate their ethical, legal and social aspects, analysing predominant technovisions and sociotechnical imaginaries can yield valuable insights into people’s explicitly stated or implicitly held values (145). Culturally shaped narratives and historically prevalent ideas about enhancing the human body can reveal problematic aspects of speculative, ethical-political discourse, as happened in the USA during the last third of the twentieth century and as is spurring a surge in neurotechnology development today (146). Prominent examples of such historically shaped imaginaries or technovisions can be found in the golems or cyborgs in the seminal works of Donna Haraway (147) that investigate body politics from a critical-feminist perspective.

Emerging neurotechnologies evoke questions about the interplay of society, technology and the individual; about human autonomy within techno-dominant structures; about the role of free will within techno-capitalist systems; and about questions of ableism and the criteria according to which human bodies are defined as healthy or enhanceable. A critical consideration of human enhancement informed by hermeneutic discourse analysis can thus help innovators and regulators understand and manage the impact of predominant technovisions; in turn, this can help researchers and developers identify notions of desirable and socially accepted forms of neurotechnologies altering human corporeality.

## 3 Discussion

As state-of-the-art neurotechnologies become increasingly accessible to a wider public, their implications may be less immediately transparent to non-specialists, limiting an intuitive grasp of their broader societal impacts. Even though societies have always adapted to technological change over the centuries and developed corresponding societal norms, the speed of innovation, the complexity of the field and the ethical sensitivity of neurotechnology may impede the development of adequate levels of neurotechnology literacy. As a result, the opportunities and limitations of these technologies might not be readily understood, similar to challenges observed in the field of artificial intelligence. Given the ethical sensitivities in neurotechnology, this lack of understanding can also hinder a balanced evaluation of both its potential benefits and risks. We are already seeing that this can lead to hype, unrealistic and oversold visions that are at odds with the outcomes of clinical research. According to the Gartner Hype Cycle, limited access to clear and accurate information can significantly bias the public view of emerging technologies. In this model, a surge of inflated expectations, the ‘hype’, is most likely to be followed by a ‘trough of disillusionment’, before applications progress from well-balanced (risk–benefit) assessments along the ‘slope of enlightenment’ toward the ‘plateau of productivity’ (148). Even though the Gartner Hype Cycle Theorem is not a definitive formula and offers only limited explanatory value for actual technology developments and innovation trajectories, it remains useful for highlighting how hypes, technofutures and socio-cultural narratives can shape the public discourse on innovation and emerging technologies (149). One effective way to limit the space in which hype can evolve and to foster more balanced public engagement is to reinforce inter- and transdisciplinary communication, both among researchers and between scientific communities and the public. While not sufficient on its own, such exchange can help promote the accurate and nuanced presentation of complex scientific and technological information. It can also help incorporate diverse perspectives and address broader social, ethical, and cultural dimensions that influence public understanding and acceptance of emerging technologies by establishing connections across different stakeholder groups and building trust (150, 151). This is particularly important as traditional format(s) of science-society communication, along with conventional engineering and medical approaches, still often tend to sideline personal experiences and psychological and cultural factors that shape the public attitude toward and acceptance of new technologies and drive or influence evolving social norms (152).

This article aims to help attribute meaning to neurotechnology, arguing that ethical, societal and regulatory discourse about neurotechnologies should more actively include patient and user perspectives in new ways. To explore this, we adopt an inter- and transdisciplinary approach that addresses a central question, namely:

- *What should be done to narrow the current gap between the emerging application of neurotechnology and the insufficient co-evolution of regulatory measures and societal norms*?

As neurotechnology becomes more accessible and is used by individuals with varying levels of neuroscientific literacy, familiarization and demystification of neurotechnology appears to be a necessary and protective step that requires public education and open, inclusive, and transparent communication.

Drawing on the context and perspectives presented in this paper, a possible strategy for addressing the central question could include: (i) providing orientation knowledge at science-society (opinion-forming) and science-policy (decision-making) interfaces; (ii) creating an agora for transdisciplinary exchange across relevant sectors; and (iii) evaluating and communicating scientific results and monitoring social negotiation processes from a critical (situated, contextual, reflexive) research perspective.

**Figure 3:**
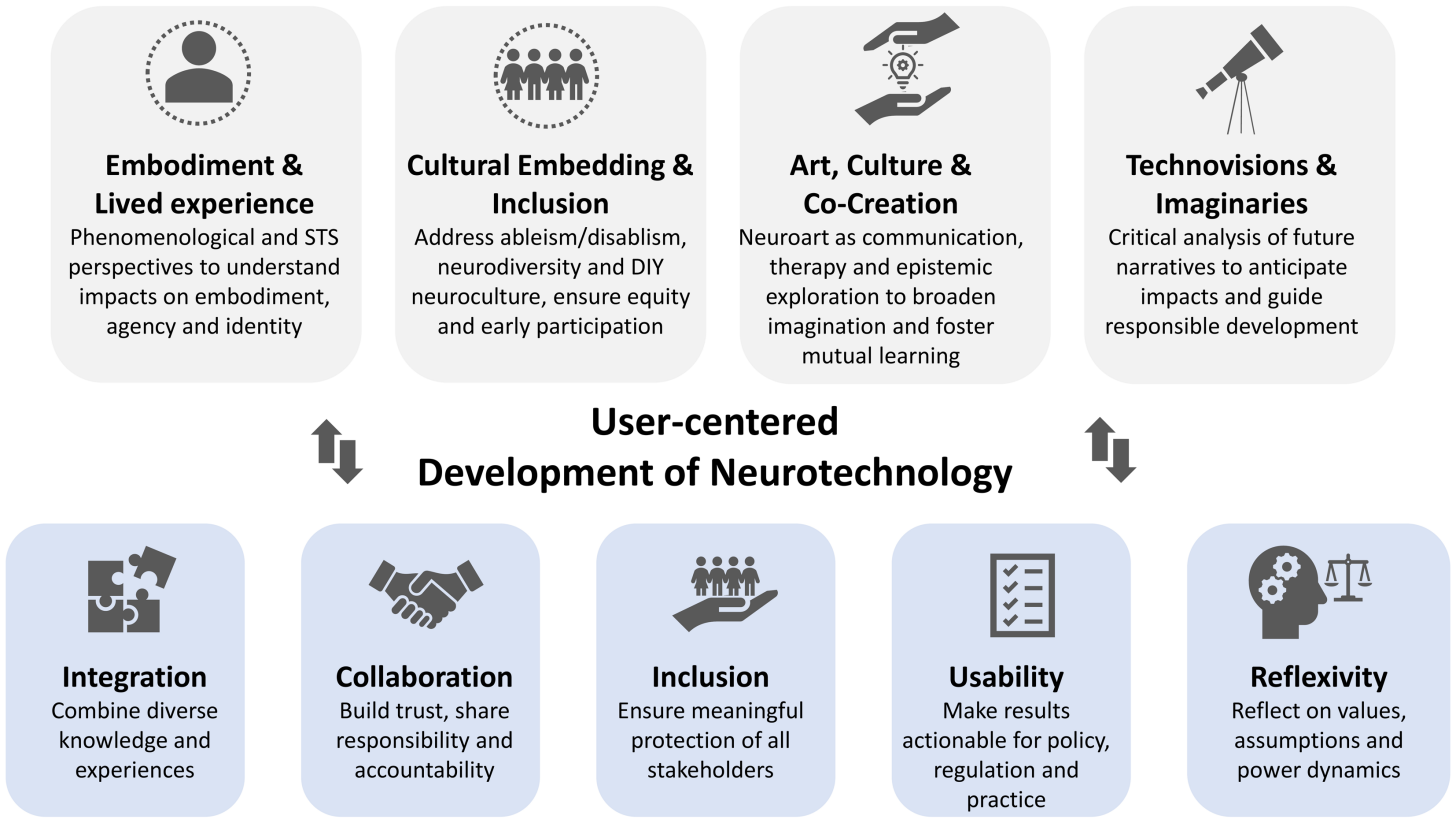
Conceptual diagram of the manuscript’s position and its approach to addressing the central question, based on the arguments presented.

As a concrete example of a research design suited to further strengthen this field, the following points interpret the criteria for good transdisciplinary research from a framework created by Merrit Polk (153), which can be applied to the socially integrated, and ‘embodied’ way of researching emerging neurotechnologies (Figure 3):

*Integration* provides platforms for the exchange, combination and emergence of new collective forms of knowledge from social actors’ diverse, inherently embodied ways of knowing. Particularly artistic and creative representations of neuroscientific ideas and practices, as well as of neurotechnology, its applications and socio-cultural implications, can serve as effective means to transparent communication and inclusive engagement with people from diverse backgrounds, including direct stakeholders in neurotechnology advancement (such as users), indirect stakeholders (e.g., funding bodies or companies) and members of the general public.

The Futurebody project (70), which included the BIO·FICTION film festival (71), is an example of such an endeavor (see Box 1 above). The project engaged a wide variety of people who deal with the human body in their daily practice, including physiotherapists, other health professionals, tattoo artists and meditation practitioners, thereby embedding or situating neurotechnological developments within a broader culture. Closely linked to these participatory activities, the project also systematically explored the visions, fears and realities around neurotechnologies from philosophical, socio-empirical and artistic perspectives. Such approaches can help not only to counter widespread illusions and uninformed hype and to promote more accurate narratives about emerging technologies, but also encourage society in all its diversity to actively engage in the discussion about the future development of neurotechnological innovations.

*Collaboration* motivates multi-stakeholder processes built on mutual trust, accountability and clearly defined responsibilities among relevant actors (users, researchers, producers, regulators etc.). Research on emerging neurotechnologies from critical, ethical and user-centered perspectives requires project teams and consortia to be carefully formed, implemented and maintained for meaningful communication and exchange, not only between researchers but also with non-academic stakeholders and social groups (154). Continuous reflections on roles and expectation management appear crucial for successful research processes and collaborations (155, 156).

*Inclusion* enables participation in social and scientific discourse, especially for affected stakeholder groups that are often marginalized or underrepresented. It is essential to recognize the limits of participatory design when potential participants face socio-economic barriers (live below the poverty line, or unemployment) and/or experience disablism burnout (systemic discrimination based on irrelevant expectations of ability (157, 158)). Disabled people, as citizens, are entitled to equal access to all applications as well as a key stakeholder group when it comes to development of prosthetic and assistive technologies. In order for them to be part of anticipatory governance, they need to engage in anticipatory advocacy (the capacity of disability rights groups to participate in science and technology governance) and thus have to be informed and involved even earlier than others in the process, since development trajectories are often already established by the time formal governance discussions take place (159). The same is true for their access to (scientific) knowledge with a view to limits of participatory designs.

*Usability* refers to designing and documenting public discourse in such a way that its results can be implemented in the technology’s legal regulation and social integration. Neuroethicists engaging in transdisciplinary research frameworks and in participatory TA need to inform, frame, guide and assess public debate in functional forms of science communication (69). Ultimately, the usability of neurotechnology should be considered from stakeholder perspectives in order for it to serve the needs of its users in a wider integrated (neuro)ethical discourse framing.

*Reflexivity* provides a communication framework that encourages active value reflection to identify bias and assumptions among all research participants. The potential of inclusive science/technology outreach, as for example in neuroart approaches, extends beyond communicative and educational gains to reap philosophical and epistemological insights. This, in turn, can strengthen the formation of a pluralistic and interdisciplinary knowledge base where ethical considerations reflect the diversity of use cases and users of neurotechnologies.

The active involvement of the diverse user base may serve as the most fundamental step toward responsible neurotechnological developments. In this context, reflexivity can take intrasubjective forms, such as personal engagement or art-based experience of neurotechnologies. Reflexivity can also support the critical examination of predominant “technovisions” and cultural narratives that shape expectations and design choices in the emerging neurotechnologies.

Recent developments give reason for cautious optimism, as growing attention is being directed toward the ethical and societal dimensions of neurotechnology. Initiatives such as the Future Foundation’s efforts to place the human being at the center – as a subject rather than a mere recipient of technological benefits – is one example of this shift in perspective (160). Similarly, the “Future Germany 2050” initiative (161) from the largest German Engineering Association VDI recommends targeted economic support to promote responsible research in neurotechnology, while UNESCO’s declaration on neurotechnology from 2025 (162), as an instrument of international soft law, aims to establish common terms and conditions between relevant stakeholders. Together, these initiatives, among many others, indicate that the wheel has begun to turn. Nevertheless, much remains to be done, and the efforts to ensure responsible governance and ethical reflection must keep pace with the rapid development of new tools and technologies.

## 4 Concluding remarks

To conclude, we emphasize that responsible innovation in neurotechnology requires more than just technical advancement, it demands ethical sensitivity, interdisciplinary collaboration and meaningful user engagement. Strengthening these dimensions can help improve the current situation in the neurotechnology landscape that appears to be negatively impacted by the asymmetry in communication between scientists and entrepreneurs on the one hand and current and potential users on the other. In this context, the underlying cause, whether ineffective communication strategies with the neurotech community or ethically questionable business or public relations practices, is secondary to the need for more transparent and inclusive dialog among all stakeholders.

Asymmetry in communication and interaction usually leads to one side feeling disempowered, excluded from decision-making, and underrepresented in regulatory discussions. In many cases, this can result in backpedaling on decisions or protests outside established structures. To counter this, the public should be provided with more equitable and symmetric communication structures and formats that empower the diverse members of involved and affected stakeholder groups, regardless of their social status. Whether healthy or otherwise, users or healthcare professionals, citizens or non-citizens, members or non-members of a public health insurance system, all should have opportunities to contribute to discourse, scenario development and decision-making on ethical and societal measures, norms and standards.

Neurotechnologies are not “simply another” therapeutic toolbox, even if they are likened to the most effective drugs. They touch on fundamental aspects of human individuality and culture. Much greater sensitivity with respect to the often radically novel character of neurotechnologically transformed corporeality and lived experience is needed. Addressing these challenges requires, on one hand, new approaches to the cultural and social embedding and understanding of neurotechnologies, including citizen science and user-centered approaches, and on the other hand, continued development of regulatory measures to ensure their applications (medical or non-medical) adhere to the highest standards of ethically responsible technology development.
